# Sociodemographic and Clinical Determinants of Glaucoma in a Sub‐Saharan African Population: A Case‐Control Study

**DOI:** 10.1002/hsr2.72259

**Published:** 2026-04-02

**Authors:** Angelina Ampong, Seth Lartey, Naa Naamuah Tagoe, Yaw Akye Essuman, Benjamin Abaidoo, Vera Mawusime Beyuo, Lauretta Pinamang Owusu‐Asare, Dziffa‐Bella Imelda‐Odille Ofori‐Adjei, Vera Adobea Essuman

**Affiliations:** ^1^ Eye Center, Komfo Anokye Teaching Hospital Adum‐Kumasi Ghana; ^2^ Lionizers International Eye Center, Korle Bu Teaching Hospital Accra Ghana; ^3^ Ophthalmology Unit, Department of Surgery University of Ghana Medical School, University of Ghana Accra Ghana

**Keywords:** adult, associations, Ghana, glaucoma, IOP, POAG, risk factors

## Abstract

**Background and Aims:**

Glaucoma is a leading cause of irreversible blindness worldwide, with a disproportionate burden in Sub‑Saharan Africa. Identifying sociodemographic and clinical determinants is critical for early detection and prevention. This study identified glaucoma risk factors in Ghanaians and evaluated their prognostic significance.

**Methods:**

A prospective hospital‐based case‐control study conducted at a tertiary hospital in Ghana from 1st October to 31st December, 2021, including 150 glaucoma patients and 150 age‐ and sex‐matched controls. Sociodemographic and clinical data were collected. Predictive risk factors were assessed via multivariate logistic regression in SPSS version 25. Statistical significance was set at *p* < 0.05.

**Results:**

Three hundred participants were recruited, including 178 (59%) females. Mean ages of glaucoma patients and controls were 58.4 ± 17.4 and 52.35 ± 18.5 years, respectively. Primary open‐angle glaucoma (POAG) was the predominant type (77.3%). Statistically significant risk factors for glaucoma were: age ≥ 40 years [OR: 3.5, 95% CI: 1.8–6.7, *p* = 0.001], positive family history [OR: 3.2, 95% CI: 1.7–5.9, *p* = 0.001], hypertension [OR: 2.7, 95% CI: 1.5–5.0, *p* = 0.002], myopia [OR: 4.3, 95% CI: 2.1–9.1, *p* = 0.001], and intraocular pressure (IOP) ≥ 22 mmHg [OR: 4.1, 95% CI: 1.9–8.9, *p* = 0.001]. Advanced glaucoma at presentation was significantly associated with IOP ≥ 22 mmHg (*p* = 0.002), myopia (*p* = 0.001), and hypertension (*p* = 0.001). POAG was significantly associated with age ≥ 40 years (*p* = 0.001), family history of glaucoma (*p* = 0.001), and education (*p* = 0.031). It was also significantly associated with IOP ≥ 22 mmHg, myopia, diabetes, and hypertension (all, *p* = 0.001).

**Conclusions:**

Age, family history of glaucoma, hypertension, myopia, and elevated IOP were key risk factors. Targeted screening and regular eye examinations among high‐risk individuals may facilitate earlier detection and help prevent or delay progressive glaucomatous optic nerve damage.

## 1 Introduction

Glaucoma is a major cause of irreversible blindness worldwide [1]. It is a group of chronic, progressive optic neuropathies that cause irreversible loss of retinal ganglion cells, resulting in consequent visual field defects and loss of visual function [2]. Glaucoma poses a major public health challenge due to its insidious onset and asymptomatic progression in early stages [3]. As a result, glaucoma has been marked as a priority eye disease by the World Health Organization (WHO), highlighting the need for region‐specific research to support prevention and management strategies for managing the condition [4]. Despite laudable advances in diagnostic and treatment modalities, a significant proportion of undiagnosed glaucoma cases remains, especially in the Global South, where access to these modalities is often limited [5].

Many sociodemographic and clinical determinants of glaucoma have been well‐established in several parts of the world [6, 7]. These include older age, race, sex, systemic comorbidities such as diabetes mellitus and hypertension, increased intraocular pressure, positive family history of glaucoma, refractive status (myopia), and thin central corneal thickness [6, 7, 8, 9, 10, 11]. Among these factors, increased intraocular pressure is the only modifiable risk factor [6]. These determinants not only influence biological susceptibility to developing glaucoma but also patients' utilization of eye care services, timing of diagnosis, and the severity of disease presentation [12]. A thorough understanding of the interplay of these clinical and social determinants is therefore integral to comprehensive glaucoma prevention and treatment interventions.

Despite the recognized importance of these factors, evidence from Sub‐Saharan African (SSA) countries, including Ghana, remains limited, as most of the existing literature on glaucoma risk factors is based on high‐income settings, where demographic profiles, anatomical features, health systems, and environmental conditions substantially differ. Moreover, there is limited empirical evidence integrating both sociodemographic and clinical determinants in our SSA populations. This gap impedes the development of targeted interventions and policies designed to ameliorate the burden of blindness due to glaucoma in the region. Existing studies in SSA primarily focused on the prevalence of glaucoma and treatment outcomes, with limited attention on the interplay between social determinants and clinical risk factors [13]. Although population‐based studies offer valuable data on glaucoma, they often lack the depth required to identify specific risk factors associated with the disease. As a relatively rare but important ocular condition with a long latency period, glaucoma is best studied through case‐control designs, which allow risk factors to be identified with smaller sample sizes while allowing researchers to retrospectively examine exposures without lengthy follow‐up times [4]. Additionally, the use of case‐control designs supports a simultaneous analysis of multiple exposures, such as sociodemographic and clinical characteristics, to provide a comprehensive understanding of the factors associated with the occurrence of the disease. The comparison of individuals diagnosed with glaucoma to those without the disease may help to explain both clinical and social determinants to inform screening and detection efforts.

This study therefore examined the risk factors for glaucoma by analyzing the sociodemographic characteristics and ocular and medical risk factors, as well as the predominant types and severity of glaucoma at the time of diagnosis among Ghanaians in the study population. We explored whether these risk factors aligned with or differed from findings in previous studies [14, 15, 16]. Additionally, this study assessed the relationships between sociodemographic, ocular, and medical risk factors and the type and severity of glaucoma, and assessed associations.

## 2 Materials and Methods

### 2.1 Study Design

This was a prospective hospital‐based case‐control study conducted at the Eye and Medicine departments of the Komfo Anokye Teaching Hospital—a tertiary healthcare facility in Kumasi, Ghana—from October 1 to December 31, 2021. We selected a case‐control design as the most appropriate design for this study, as it balances efficiency, feasibility, and depth of analysis, allowing the study to uncover both social and clinical determinants of glaucoma without requiring prohibitively large or long‐term studies.

### 2.2 Study Setting

The Komfo Anokye Teaching Hospital was chosen as the study setting because it serves as a major referral center for diverse patients from several parts of Ghana. Recruiting both cases and controls from this hospital ensured feasibility, minimized the level of bias, and provided a representative sample for analyzing the determinants of glaucoma in the regional population.

### 2.3 Study Population

Our study population comprised adult patients aged 18 years and above with (case) and without (controls) glaucoma who attended the Eye and Medicine departments of the Komfo Anokye Teaching Hospital during the study period.

### 2.4 Definition of Cases and Controls

Cases were individuals selected from the Eye Department and diagnosed with glaucoma according to the International Society of Geographical and Epidemiological Ophthalmology (ISGEO) criteria. Controls were adults without clinical evidence of glaucoma, matched to cases by age and sex, and recruited from the Department of Medicine in the same hospital.

For the cases, we included adults aged ≥ 18 years diagnosed with glaucoma based on ISGEO definitions as: participants with evidence of glaucomatous optic neuropathy (vertical cup‐to‐disc ratio ≥ 0.7 or asymmetry ≥ 0.2 between eyes) with corresponding visual field loss, advanced structural optic nerve damage (vertical cup‐to‐disc ratio ≥ 0.9) in cases where visual field testing was not possible, and participants with severe visual impairment (visual acuity < 3/60) with intraocular pressure ≥ 99th percentile for the population, in cases where optic disc assessment was not feasible. We excluded participants with secondary glaucoma due to conditions such as trauma, uveitis, or the use of steroids. We also excluded participants with ocular conditions that could mimic glaucomatous optic neuropathy (e.g., optic neuritis, ischemic optic neuropathy) and patients with media opacities (such as dense cataracts) that precluded adequate optic disc examination or visual field testing.

For the controls, we included adults without clinical evidence of glaucoma, matched by age and sex to cases, and excluded participants younger than 18 years and those with optic disc or other ocular examination findings suspicious for glaucoma. Suspicious findings included a cup‑to‑disc ratio (CDR) greater than 0.4, or inter‑eye CDR asymmetry greater than 0.2, consistent with ISGEO recommendations to minimize misclassification and ensure that controls truly represented non‑glaucomatous eyes. We excluded controls under 18 years old with optic disc or other ocular findings suggestive of glaucoma to avoid misclassification, ensure diagnostic accuracy, and maintain study validity. Including such participants could confound results, as early or secondary glaucomatous changes might bias comparisons between true cases and healthy controls.

### 2.5 Sample Size Determination

For this matched case‐control study, the formula [17] below was used:

N = (r + 1) [1 + (λ−1) P] 2/rP2 (P‐1) 2(λ−1) 2[Zα/2 √ (r + 1) Pc + Zβ√ ({λP (1‐P)/(1 + (λ−1)P) 2 +rP (1‐P)] 2. Pc = P/(r + 1) [rλ/1+ (λ−1) P + 1],

where p and λ are the prevalence of glaucoma (6.8%) [18] and risk of family history of glaucoma among glaucoma patients (3.08) from a previous study [19], r is ratio of cases to control (1:1), Zβ is the desired power (typically 0.84 for 80% power), and Zα/2 is the desired level of statistical significance (typically 1.96). From the above formula, the required sample size was *n* = 290. A simple random sampling technique was used to select 300 consenting patients who met the inclusion criteria, comprising 150 cases and controls each. We estimated the sample size using the prevalence of family history of glaucoma from a previous study, as it is a well‑established, measurable determinant, providing a reliable basis despite multiple risk factors being explored.

### 2.6 Study Procedure

A sampling frame, consisting of the appointment schedules for newly diagnosed participants with glaucoma, was obtained from the general outpatient clinic. The folder identification numbers (IDs) of the glaucoma patients who met the inclusion criteria were entered into a Microsoft Excel 2015 worksheet. A command was given for the randomization of the list of IDs provided via a random number generator (RAND function) in Microsoft Excel 2015 software. The first five IDs to appear were selected, and the patients were invited to participate in the study. This was done on each glaucoma clinic day, run twice a week, and at the general medicine clinic until the desired sample size was attained. The same sampling method was used in the sampling of the controls, who were matched for age and sex.

### 2.7 Glaucoma Classification and Staging

The type/classification of glaucoma for each subject was determined by the PI as “primary” or “secondary” and “open‐angle” or “closed‐angle” using the presence or absence of other ocular abnormalities that could be responsible for the glaucoma, and the gonioscopy findings, respectively.

Staging of glaucoma for each eye was performed by the PI via the International Classification of Diseases 10th Edition (ICD‐10) [20], staging of glaucoma as follows:

1. Mild/early glaucoma: Optic disc findings consistent with glaucoma with normal visual fields/no visual field defect and focal thinning of the retinal nerve fiber layer on OCT.
2. Moderate glaucoma: Optic disc findings consistent with glaucoma with visual field abnormalities in one hemifield but not within 5 degrees of fixation.
3. Severe glaucoma: Optic disc findings consistent with glaucoma with visual field abnormalities in both hemifields and/or loss within 5 degrees of fixation in one hemifield.
4. Indeterminate glaucoma: Optic disc abnormalities consistent with glaucoma with no visual field data.

Each eye was staged separately, that is, the unit of staging was individual eyes and not study subjects. When visual field testing was not possible (due to very poor visual acuity and/or inability to fixate for the test to be performed) or was not reliable, the diagnosis of glaucoma was made based on optic nerve findings and gonioscopy.

### 2.8 Data Collection

Patient sociodemographic details, including age, sex, address, occupation, and highest educational level attained, were documented via a pretested, structured questionnaire. The presenting complaints of each subject were recorded on the data collection form. The clinical characteristics of the participants, including ocular history (history of ocular trauma, ocular surgery, and spectacle use), family history of glaucoma, age at onset of glaucoma, and duration of glaucoma, were documented. Histories of systemic conditions such as diabetes mellitus, hypertension, migraine, and sickle cell disease were obtained verbally and recorded.

The presenting distance visual acuity was measured by an assigned, trained ophthalmic nurse, via a LogMAR distance visual acuity chart, with the participant seated 6 m away from the chart and one eye tested at a time while the other eye was occluded. The visual acuity status of each subject was classified according to the International Classification of Diseases 10th Edition (ICD‐10) [20], as normal vision (> 0.3), mild visual impairment (< 0.3–0.5), moderate visual impairment (< 0.5–1.0), severe visual impairment (< 1.0–1.3), and blindness (< 1.3). Visual acuity data for each eye were used separately for analysis.

Anterior segment examination was performed using slit lamp biomicroscopy to determine the clarity of the cornea, status of the lens, anterior chamber depth, and iris configuration. We measured the intraocular pressures of participants with the Goldmann applanation tonometer mounted on a slit lamp biomicroscope following the instillation of 0.5% proparacaine and fluorescein dye into the conjunctival sac of participants' eyes. Following standard calibration procedures, the tonometer prism was gently positioned at the center of the cornea with the slit lamp illumination and alignment adjusted until the semicircular mires appeared visible. Readings of the IOP were then recorded for both eyes. We also performed gonioscopy to assess the anterior chamber angle using a Goldmann three‐mirror lens. This was done by initially instilling 0.5% amethocaine eye drops, followed by the placement of the gonioscopy lens on the cornea with coupling fluid. Angle structures were then examined systematically in all quadrants. Findings were then documented and classified according to Shaffer's grading system [21]. We dilated the fundus using a combination of topical tropicamide 1% and phenylephrine 2.5% and performed posterior segment examination using a 90D lens with the slit lamp or a direct ophthalmoscope. Optic disc morphology was assessed, including cup‐to‐disc ratio (CDR), neuroretinal rim integrity, and presence of glaucomatous changes. The macula and peripheral retina were also examined for coexisting pathologies.

### 2.9 Statistical Analysis

Statistical analysis was performed via IBM SPSS version 25 (IBM Corp., Armonk, NY, USA). For the sociodemographic and clinical characteristics, categorical variables were summarized as frequencies and percentages, while continuous variables were expressed as means with standard deviations. We assessed normality of continuous variables using the Shapiro–Wilk test, and data were summarized as mean ± standard deviation for normally distributed variables or median (interquartile range: IQR) for non‐normally distributed variables. Multivariate logistic regression was used to analyze the predictive risk factors for glaucoma. Odds ratios (OR) and 95% confidence intervals (95% CI) were presented. A two‐sided chi‐square test was used to assess significant differences between the characteristics of cases and controls, as well as the associations between sociodemographic and clinical risk factors and types of glaucoma. Statistical significance was set at *p* < 0.05.

### 2.10 Ethical Considerations

Informed, written consent was obtained from the participants. Illiterate patients provided consent by affixing their thumbprint after a detailed explanation of the study was provided. The study protocol (No. KATH IRB/AP/116/21) was approved by the Institutional Ethics Committee of the Komfo Anokye Teaching Hospital. This research adhered to the tenets of the Declaration of Helsinki and followed STROBE guidelines in presenting our methodology.

## 3 Results

### 3.1 Sociodemographic and Clinical Characteristics of the Study Participants

The sociodemographic and clinical characteristics of the participants are presented in Table 1. A total of 150 cases and 150 controls were recruited into the study. The mean age of the cases was 58.4 ± 17.4 years with an age range of 18 to 93 years. The mean age of the controls was 52.35 ± 18.5 years with an age range of 18 to 93 years. An analysis of variance (ANOVA) revealed a significant difference between cases and controls in mean age (*p* = 0.004; F = 8.46). Significant differences between cases and controls were observed for the following parameters: age, occupational status, family history of glaucoma, hypertension, diabetes mellitus, refractive errors in either eye, central corneal thickness in either eye, and mean intraocular pressure bilaterally (all, *p* < 0.05).

**Table 1:** Sociodemographic and clinical characteristics of adults with glaucoma at the Komfo Anokye Teaching Hospital (*N* = 300).

Variable	Cases *N* (%)	Control *N* (%)	*p*‐value
Mean age, years†	58.4 ± 17.4	52.35 ± 18.5	
Age, years:			0.004*
< 40	20 (24.4)	62 (75.6)	
≥ 40	130 (59.6)	88 (40.4)	
Sex			> 0.99
Male	61 (40.7)	61 (40.4)	
Female	89 (59.3)	89 (59.3)	
Educational level			0.140
None	52 (17.33)	41 (13.7)	
Basic	40 (13.3)	32 (10.7)	
Secondary	21 (7.0)	33 (11.0)	
Tertiary	37 (12.3)	44 (14.7)	
Occupation			0.019*
Employed	120 (40.0)	108 (36.0)	
Unemployed	30 (10.0)	42 (14.0)	
Family history of glaucoma	65 (21.7)	26 (8.7)	0.001*
Hypertension	75 (50.0)	35 (23.3)	0.001*
Diabetes mellitus	24 (16.0)	11 (7.3)	0.030*
History of migraine	4 (2.7)	4 (2.7)	> 0.99
Sickle cell disease	1 (0.7)	1 (0.7)	> 0.99
Refractive error, OD			0.001*
Myopia	31 (10.3)	3 (1.0)	
Hyperopia	10 (3.3)	15 (5.0)	
Refractive error, OS			0.001*
Myopia	32 (10.7)	3 (1.0)	
Hyperopia	11 (3.7)	16 (5.3)	
Right eye central corneal thickness, (µm)†	517.3 ± 35.3	539.8 ± 30.2	0.002*
Thin central cornea (≤ 533)	103 (68.7)	76 (50.7)	
Thick central cornea (> 533)	47 (31.3)	74 (49.3)	
Left eye central corneal thickness, (µm)†	517.1 ± 32.8	540.1 ± 29.8	0.001*
Thin central cornea (≤ 533)	106 (70.7)	76 (50.7)	
Thick central cornea (> 533)	44 (29.3)	74 (49.3)	0.001*
Right eye mean intra ocular pressure, (mmHg)†	33.2 ± 10.1	21.9 ± 3.8	0.001*
Left eye mean intra ocular pressure, (mmHg)†	33.6 ± 10.5	21.8 ± 3.8	0.006*

*Note:* OS, Oculus sinister (Left eye), OD, Oculus dexter (Right eye); Analysis of variance (ANOVA) (*p*‐value = 0.004; F = 8.46).

* Statistically significant associations.

^†^ mean ± standard deviation.

### 3.2 Ocular Examination Findings in Cases

The findings from the ocular examination of cases in this study are shown in Table 2. Most cases had open angles (OS: 132/150, 88.0%; OD: 128/150, 85.3%). Most cases had moderate to severe optic nerve damage in the left eye (122/150, 81.3%) and right eye (119/150, 79.3%).

**Table 2:** Clinical characteristics of adults with glaucoma at the Komfo Anokye Teaching Hospital (*N* = 150).

Clinical features	Frequency	Percentage (%)
Right gonioscopy findings		
Angle not visible	4	2.7
Open	128	85.3
Closed/narrow	18	12.0
Left gonioscopy findings		
Angle not visible	—	—
Open	132	88.0
Closed/narrow	18	12.0
Level of optic nerve damage, OD**		
No optic nerve damage	3	3.4
Mild optic nerve damage	20	13.3
Moderate to severe optic nerve damage	119	79.3
Optic disc not visible	6	4.0
Level of optic nerve damage, OS**		
No optic nerve damage	5	4.7
Mild optic nerve damage	19	12.7
Moderate to severe optic nerve damage	122	81.3
Optic disc not visible	2	1.3
Visual fields defects, OD		
Visual fields test not done/possible	21	14.0
No visual fields defect	14	9.4
Visual fields defect in one hemifield outside 5 degrees offixation	41	27.3
Visual fields defect in both hemifields with defect in 1 or both hemifields within 5 degrees of fixation	74	49.3
Visual fields defects, OS		
Visual fields test not done/possible	11	7.3
No visual fields defect	18	12.0
Visual fields defect in one hemifield outside 5 degrees offixation	37	24.7
Visual fields defect in both hemifields with defect in 1 or both hemifields within 5 degrees of fixation	84	56.0
Severity of glaucoma, OD		
Mild	12	8.0
Moderate	43	28.7
Advanced/severe	76	50.7
Indeterminate	19	12.7
Severity of glaucoma, OS		
Mild	12	8.0
Moderate	40	26.7
Advanced/severe	87	58.0
Indeterminate	11	3.7

Abbreviations: ODl, Oculus dexter (Right eye); OS, Oculus sinister (Left eye).

** Two values are missing.

### 3.3 Univariate Analysis of Risk Factors for Glaucoma

Table 3 shows the univariate analysis of risk factors for glaucoma. Age ≥ 40 years [OR: 4.6, 95% CI: 2.6–8.1, *p* = 0.001], family history of glaucoma [OR: 3.6, 95% CI: 2.1–6.2, *p* = 0.001], history of hypertension [OR: 3.3, 95% CI: 2.0–5.4, *p* = 0.001], history of diabetes mellitus [OR: 2.4, 95% CI: 1.1–5.1, *p* = 0.03], myopia [OR: 4.2, 95% CI: 2.2–8.0, *p* = 0.001], CCT ≤ 533 µm [OR: 2.1, 95% CI: 1.3–3.4, *p* = 0.001], and IOP ≥ 22 mmHg [OR: 3.2, 95% CI: 1.6–6.3, *p* = 0.001] were identified as predictive risk factors for glaucoma. Participants aged 40 years and above were 4.6 times more likely to have glaucoma than those younger than 40 years. Participants with a positive family history of glaucoma were 3.6 times more likely to have glaucoma than participants with no family history of glaucoma.

**Table 3:** Univariate analysis of risk factors in adults with glaucoma at the Komfo Anokye Teaching Hospital.

Risk factors	Odds ratio	95% CI	*p*‐value
Age, years			
< 40	Reference	2.6–8.1	0.001*
≥ 40	4.6		
Sex			
Female	Reference	0.6–1.6	0.547
Male	1.0		
Educational status			
Educated	Reference		
Uneducated	0.7	0.4–1.2	0.212
Occupation			
Unskilled	Reference	0.5–1.2	0.239
Skilled	0.7		
Family history of glaucoma	3.6	2.1–6.2	0.001*
Presence of comorbid conditions			
Hypertension	3.3	2.0–5.4	0.001*
Diabetes mellitus	2.4	1.1–5.1	0.03*
Migraine	1.0	0.2–4.1	0.639
Sickle cell disease	1.0	0.1–16.1	0.751
Presence of refractive errors			
Myopia	4.2	2.2–8.0	0.001*
Hyperopia	0.6	0.3–1.5	0.420
Central corneal thickness, µm			
> 533	Reference		
≤ 533	2.1	1.3–3.4	0.001*
Intraocular pressure, mmHg			
< 22	Reference		
≥ 22	3.2	1.6–6.3	0.001*

Abbreviation: CI, confidence interval.

* Statistically significant associations.

### 3.4 Multivariate Logistic Regression Analysis of Risk Factors for Glaucoma

Table 4 illustrates the multivariate logistic regression analysis of risk factors for glaucoma. The following were identified as predictive risk factors for glaucoma: age ≥ 40 years [OR: 3.5, 95% CI: 1.8–6.7, *p* = 0.001], family history of glaucoma [OR: 3.2, 95% CI: 1.7–5.9, *p* = 0.001], history of hypertension [OR: 2.7, 95% CI: 1.5–5.0, *p* = 0.002], myopia [OR: 4.3, 95% CI: 2.1–9.1, *p* = 0.001], and IOP ≥ 22 mmHg [OR: 4.1, 95% CI: 1.9–8.9, *p* = 0.001].

**Table 4:** Multivariate logistic regression analysis of risk factors in adults with glaucoma at the Komfo Anokye Teaching Hospital.

Risk factors	Odds ratio	95% CI	*p*‐value
Age, years			
< 40	Reference		
≥ 40	3.5	1.8–6.7	0.001*
Family history of glaucoma	3.2	1.7–5.9	0.001*
Hypertension	2.7	1.5–5.0	0.002*
Diabetes mellitus	1.7	0.7–4.4	0.233
Myopia	4.3	2.1–9.1	0.001*
Central corneal thickness, µm			
> 533 (thick)	Reference		
≤ 533 (thin)	1.3	0.7–2.3	0.393
Intraocular pressure, mmHg			
< 22	Reference		
≥ 22	4.1	1.9–8.9	0.001*

Abbreviation: CI, confidence interval.

* Statistically significant associations.

### 3.5 Associations Between Sociodemographic and Clinical Risk Factors and the Types of Glaucoma

Table 5 reveals that primary open‐angle glaucoma (POAG) was the most prevalent type of glaucoma, contributing to 77.3% (116/150) of all types of glaucoma in the study. Statistically significant associations were found between the type of glaucoma and the following patient characteristics: age ≥ 40 years, having a formal education, family history of glaucoma, intraocular pressure ≥ 22 mmHg, myopia, diabetes, and hypertension (all, *p* < 0.05).

**Table 5:** Associations between sociodemographic and clinical risk factors and types of glaucoma in adults with glaucoma at the Komfo Anokye Teaching Hospital.

Risk factors	Type of glaucoma						*p*‐value
	POAG *N* (%)	PACG *N* (%)	SOAG *N* (%)	SACG *N* (%)	NTG *N* (%)	Total *N* (%)	
Age, years							
< 40	18 (12.0)	—	1 (0.7)	—	1 (0.7)	20 (13.3)	—
≥ 40	98 (65.3)	12 (8.0)	4 (2.7)	8 (5.3)	8 (5.3)	130 (86.7)	0.001*, *
Sex							
Female	65 (43.3)	11 (7.3)	2 (1.3)	4 (2.7)	7 (4.6)	89 (59.3)	0.153
Male	51 (34.0)	1 (0.7)	3 (2.0)	4 (2.7)	2 (1.3)	61 (40.7)	—
Educational status							
Educated	78 (52.0)	6 (4.0)	4 (4.0)	2 (1.3)	8 (5.3)	98 (65.3)	0.031*, *
Uneducated	38 (25.3)	6 (4.0)	1 (0.7)	6 (4.0)	1 (0.7)	52 (34.7)	—
Occupation							
Skilled	43 (28.7)	4 (2.7)	1 (0.7)	2 (1.3)	5 (3.3)	55 (36.7)	0.535
Unskilled	73 (48.7)	8 (5.3)	4 (2.7)	6 (4.0)	4 (2.7)	95 (63.3)	—
Family history of glaucoma	50 (33.3)	6 (4.0)	1 (0.7)	4 (2.7)	4 (2.7)	65 (43.3)	0.001*, *
Refractive status							
Myopia	36 (24.0)	1 (0.7)	3 (2.0)	2 (1.3)	3 (2.0)	45 (30.0)	< 0.001*, *
Hyperopia	7 (4.7)	2 (1.3)	—	—	2 (1.3)	11 (7.3)	0.324
Intraocular pressure, mmHg							
< 22	5 (3.3)	—	—	1 (0.7)	7 (4.7)	13 (8.7)	
≥ 22	111 (74.0)	12 (8.0)	5 (3.3)	7 (4.7)	2 (1.3)	137 (91.3)	< 0.001*, *
Central corneal thickness, µm							
≤ 533 (thin)	85 (56.7)	6 (4.0)	4 (2.7)	3 (2.0)	5 (5.3)	103 (68.7)	0.102
> 533 (thick)	31 (20.7)	6 (4.0)	1 (0.7)	5 (3.3)	4 (2.7)	47 (31.3)	—
Presence of comorbid conditions							
Hypertension	58 (38.7)	9 (6.0)	2 (1.3)	4 (2.7)	2 (1.3)	75 (50.0)	< 0.001*, *
Diabetes mellitus	18 (12.0)	6 (4.0)		—		24 (16.0)	< 0.001*, *

Abbreviations: *N*, number; NTG, normal tension glaucoma; PACG, primary angle closure glaucoma; POAG, primary open angle glaucoma; SACG, secondary angle closure glaucoma; SOAG, secondary open angle glaucoma.

* Statistically significant associations.

There was no association between sociodemographic risk factors and the severity of glaucoma (*p* > 0.05). However, some clinical factors, including intraocular pressure, myopia, hypertension, and family history of glaucoma, were all associated with the severity of glaucoma (*p* < 0.05) (Table 6).

**Table 6:** Associations between sociodemographic and clinical risk factors and disease severity in adults with glaucoma at the Komfo Anokye Teaching Hospital.

Risk factors	Severity of glaucoma						*p*‐value
	None	Mild	Moderate	Severe	Indeterminate	Total	
Age, years							
< 40	1 (0.6)	2 (1.3)	6 (4.0)	8 (5.3)	3 (2.0)	20 (13.3)	—
≥ 40	2 (1.3)	10 (6.7)	35 (23.3)	67 (44.7)	16 (10.7)	130 (86.7)	0.777
Sex							
Female	2 (1.3)	6 (4.0)	25 (16.7)	43 (28.7)	13 (8.7)	89 (59.3)	—
Male	1 (0.7)	6 (4.0)	16 (10.7)	32 (21.3)	6 (4.0)	61 (40.7)	0.858
Educational status							
Educated	2 (1.3)	8 (5.3)	32 (21.3)	48 (32.0)	8 (5.3)	98 (65.3)	0.111
Uneducated	1 (0.7)	4 (2.7)	9 (6.0)	27 (18.0)	11 (7.3)	52 (34.7)	—
Occupation							
Skilled	—	4 (2.7)	18 (12.0)	30 (20.0)	3 (2.0)	55 (36.7)	—
Unskilled	3 (2.0)	8 (5.3)	23 (15.3)	45 (30.0)	16 (10.7)	95 (63.3)	0.156
Refractive status							
Myopia	—	4 (2.7)	11 (7.3)	26 (17.3)	4 (2.7)	45 (30.0)	< 0.001*, *
Hyperopia	—	—	5 (3.3)	6 (4.0)	—	11 (7.3)	0.387
Intraocular pressure, mmHg							
< 22	—	3 (2.0)	6 (4.0)	3 (2.0)	1 (0.7)	13 (8.7)	—
≥ 22	—	9 (6.0)	36 (24.0)	74 (49.3)	18 (12.0)	137 (91.3)	0.002*, *
Central corneal thickness, µm							
≤ 533 (thin)	—	7 (4.7)	28 (18.7)	56 (37.3)	12 (8.0)	103 (68.7)	0.675
> 533 (thick)	—	5 (3.3)	14 (9.3)	21 (14.0)	7 (4.7)	47 (31.3)	—
Presence of co‐morbid conditions							
Hypertension	—	8 (5.3)	18 (12.0)	38 (25.3)	11 (7.3)	75 (50.0)	< 0.001*, *
Diabetes mellitus	—	2 (1.3)	7 (4.7)	13 (8.7)	2 (1.3)	24 (16.0)	0.192
Family history of glaucoma	—	4 (2.7)	11 (7.3)	40 (26.7)	10 (6.7)	65 (43.3)	0.037*, *

* There was no association between sociodemographic risk factors and the severity of glaucoma (*p* > 0.05). However, some clinical factors, including intraocular pressure, myopia, hypertension, and family history of glaucoma, were all associated with the severity of glaucoma (*p* < 0.05).

## 4 Discussion

Glaucoma is a complex disease influenced by multiple risk factors, including elevated intraocular pressure (IOP), aging, myopia, family history, and thin central corneal thickness (CCT) [22]. Clinical and sociodemographic risk factors have been described in the literature, especially with respect to advanced glaucoma [16, 23]. This study sought to evaluate risk factors among black Africans more broadly, compare the findings with those in the literature, and assess the prognostic significance of these factors.

A multivariate logistic regression analysis of risk factors for glaucoma showed that increasing age (≥ 40 years) [OR: 3.5, 95% CI: 1.8–6.7, *p* = 0.001], positive family history of glaucoma [OR: 3.2, 95% CI: 1.7–5.9, *p* = 0.001], history of hypertension [OR: 2.7, 95% CI: 1.5–5.0, *p* = 0.002], myopia [OR: 4.3, 95% CI: 2.1–9.1, *p* = 0.001], and IOP ≥ 22 mmHg [OR: 4.1, 95% CI: 1.9–8.9, *p* = 0.001] were risk factors for glaucoma.

This study revealed that primary open‐angle glaucoma (POAG) was the most common type of glaucoma, accounting for 77% of cases in this study. This number is greater than that reported in India [2], but findings corroborate data that indicate that it is the most common type of glaucoma in African populations [15]. We observed advanced presentations in half of our cases (50.0%). In this study, advanced glaucoma at presentation was significantly associated with an IOP ≥ 22 mmHg, myopia, and systemic hypertension. Another study in Ghana reported that high intraocular pressure at presentation, ethnicity, and age > 60 years were predictors of late presentation of primary open‐angle glaucoma [24]. Though the mean age of glaucoma cases in our study was 58.4 years, which is similar to that of the aforementioned study, our study did not find advanced age to be associated with late presentation of POAG. This may be attributed to the fact that many individuals with glaucoma remain unaware of their condition because of its largely asymptomatic nature in the early stages. Consequently, it is common for patients to seek medical attention only after significant glaucomatous damage and visual impairment or blindness have already occurred [16, 25].

The mean ages of the cases and controls were 58.4 ± 17.4 and 52.35 ± 18.5 years, respectively. This is greater than findings described in other parts of Ghana, where the mean age was 54 years in both cases and controls [26]. The difference may be explained by the difference in study design, where the current study was hospital‐based and that of Ntim‐Amponsah et al was population‐based [26]. Ageing is postulated to affect glaucoma progression by resulting in greater retinal nerve fiber layer loss at similar intraocular pressure to younger patients [27]. There may also be associated biomechanical changes in the optic nerve head with ageing, as well as mitochondrial dysfunction and vascular alterations [28].

Among the 150 cases, females comprised 59.3%, and 40.7% were males. The proportion of males is lower than 67.3% [23], and 65.4% [2] observed in studies from India, and 63.8% [16] in Ethiopia. The female preponderance is, however, similar to findings from the Rotterdam Study [29]. This female predominance among cases could be explained by theories suggesting that women not only live longer than men but also have a higher prevalence of POAG worldwide [30]. Although no definitive gender preference has been established for open‐angle glaucoma, females are at a greater risk for angle‐closure glaucoma [31]. Some research suggests that female sex hormones may have a protective effect on the optic nerve; thus, with aging, a decrease in estrogen exposure has been hypothesized to increase the risk of POAG, although findings from population‐based studies remain inconsistent [29]. Women appear to bear a greater burden of glaucoma‐related blindness, which may be attributed to their longer life expectancy and socioeconomic or health‐related disparities [31]. There was no significant difference in sex between the cases and controls (*p* > 0.99).

We found that patients with a positive family history of glaucoma had a threefold increased risk of developing glaucoma [OR: 3.2, 95% CI: 1.7–5.9, *p* = 0.001] and a significant risk of developing POAG. These findings attest to the heritability of glaucoma, as there is a 22% lifetime risk of glaucoma among first‐degree relatives of glaucoma patients as compared to a 2.3% risk in those without a positive family history of glaucoma [32]. We also found a significant association between a positive family history of glaucoma and severe glaucomatous disease. This was also similar to the findings of other studies that reported that a positive family history was associated with a fivefold greater risk of developing advanced disease [16]. Other studies found that patients who did not have a family history of glaucoma had a two to threefold greater risk of presenting with advanced disease than those with a positive family history [22, 24]. This can be explained by the fact that a patient with a family history of glaucoma is more likely to undergo periodic screening, leading to an earlier diagnosis. In contrast, an individual without a family history may be less aware of the condition and, therefore, more likely to be diagnosed at a later stage. Although we expect patients with a positive family history of glaucoma to have greater awareness, several contextual factors explain why they are still diagnosed at an advanced stage and remain untreated. In our SSA populations, limited access to eye care services, low health‑seeking behavior, financial constraints, and lack of routine screening programs often delay the diagnosis of glaucoma. As a result, awareness of risk may not necessarily translate into preventive action, particularly where ophthalmic services are scarce or concentrated in urban centers. Additionally, since glaucoma is an asymptomatic condition in its early stages, family members may not recognize the need for screening until vision loss becomes significant. The prevalence of cardiovascular comorbidities, such as hypertension (*p* = 0.001) and diabetes mellitus (*p* = 0.03), was significantly higher in cases than in controls. Systemic hypertension frequently coexists with glaucoma, possibly due to the dysregulation of blood flow that characterizes both diseases [33]. We found a significant association between systemic hypertension and the severity of glaucoma (*p* < 0.001). A recent meta‐analysis [34] revealed a significant association between diabetes mellitus and glaucoma, with proposed underlying mechanisms including compromised neuronal and glial function leading to the apoptosis of retinal ganglion cells and increased synthesis and accumulation of fibronectin in the trabecular meshwork, resulting in impaired outflow [35, 36].

Myopia was found to be significantly associated with the severity of glaucoma (*p* < 0.001) and POAG (*p* < 0.001). It was a significant risk factor for glaucoma on both univariate and multivariate analysis (*p* < 0.001). Myopia has been described as a risk factor for glaucoma in several studies [2, 37, 38]. Myopic eyes are believed to be more susceptible to IOP‐induced damage due to abnormal organization of connective tissue, with a recent meta‐analysis identifying a 20% increased glaucoma risk with each unit increase in myopia [39, 40]. This finding in a Ghanaian population is of particular interest as myopia has been described more frequently as a prominent risk factor in Asian populations [41].

The role of increased intraocular pressure in the progression has been well‐documented in many studies [42, 43]. Multiple mechanisms underlying this causal relationship have been postulated, including direct axonal damage to the retinal ganglion cells [44], ischemic damage to the optic nerve head [45], and impaired metabolic supply of the optic nerve head from the astrocytes [46]. We found that increasing intraocular pressure (≥ 22 mmHg) was associated with types of glaucoma (*p* < 0.001) and severity of glaucoma (*p* = 0.002). This finding is similar to those of other studies that have described IOP > 25 mmHg as a risk factor for advanced disease [16, 22, 47]. High intraocular pressure at presentation, as well as an increase of 1 mmHg above 25 mmHg, was associated with increased risk in another study [24].

The majority of the cases (68.7%) had a thin central cornea (≤ 533 µm) in the right eye, while 50.7% of the controls also had a thin cornea. Similarly, 70.7% of the cases had a thin central cornea (≤ 533 µm) in the left eye, whereas 50.7% of the controls did. The mean central corneal thickness (CCT) of the right eye in the cases (517.3 ± 35.3 µm) was significantly lower than in the control group (539.8 ± 30.2 µm). Similarly, the mean CCT of the left eye in the cases (517.1 ± 32.8 µm) was significantly lower (*p* = 0.001) than in the control group (540.1 ± 29.8 µm). Thin corneas in this population were consistent with findings from another study in a Ghanaian population [48]. However, unlike the present study, which revealed a statistically significant difference in CCT between cases and controls, Ntim‐Amponsah et al. did not find a significant difference in the right eye but reported a weakly significant difference in the left eye [48]. Other studies have described thin corneas in Africans and people of African descent [37, 38, 49]. One hypothesis is that a lower CCT may be associated with weaker collagen in the sclera, cornea, and lamina cribrosa (LC) [50]. The structural integrity of the sclera is crucial in determining how the lamina cribrosa responds to pressure‐induced deformation and, consequently, damage to the optic nerve. Patients with lower corneal deformation amplitudes tend to have greater lamina cribrosa depths, larger cup areas, deeper optic cups, and smaller peripapillary atrophy zones than those with higher corneal deformation amplitudes [50]. This thin cornea makes the optic nerve vulnerable to glaucomatous damage [22].

Among the 228 employed participants, 52.6% were cases and 47.4% were controls. There was a statistically significant difference in occupational status between cases and controls (*p* = 0.019); however, this was not observed in other studies [51]. We did not find a statistically significant difference in education between the cases and controls either (*p* = 0.141). This aligns with the findings of some authors [47, 51]. We also found no association between sociodemographic risk factors and the severity of glaucoma (*p* > 0.05). Although some studies have reported a significant association between migraines and glaucoma [52, 53], we detected no significant difference between cases with migraine and controls with migraine (*p* > 0.99). This may be due to the small number of participants with migraines, with only four cases and four controls affected.

A limitation of the current study is that it was a single‐center study and would have benefited from involving other facilities in the country to get a more accurate picture of the glaucoma burden in Ghana. It, however, forms a foundational basis for future studies in this area. Another possible limitation was that blood pressure and blood glucose measurements were not taken, creating the possibility of undiagnosed patients. Further studies on this subject in Ghana and Africa should, thus, aim for a multicenter study design incorporating objective assessment of cardiovascular diseases.

## 5 Conclusion

The study reveals glaucoma is caused by a multifactorial etiology, with age, family history, hypertension, myopia, and elevated intraocular pressure being significant risk factors. Delayed diagnosis or inadequate management may accelerate disease progression. Targeted screening strategies, early detection, and integrated management of systemic conditions can mitigate glaucoma‐related vision loss. Public health initiatives should therefore prioritize education and awareness.

## Author Contributions

**Angelina Ampong:** conceptualization, investigation, data curation, writing – original draft, writing – review and editing. **Seth Lartey:** conceptualization, data curation, investigation, writing – review and editing, supervision. **Naa Naamuah Tagoe:** conceptualization, writing – draft, writing ‐ review and editing, supervision. **Yaw Akye Essuman:** writing – review and editing, methodology, project administration. **Benjamin Abaidoo:** formal analysis, writing – review and editing; supervision, data curation, methodology, software. **Vera Mawusime Beyuo:** writing ‐ review and editing. **Lauretta Pinamang Owusu‐Asare:** data curation, writing – review and editing. **Dziffa‐Bella Imelda‐Odille Ofori‐Adjei:** writing – review and editing. **Vera Adobea Essuman:** writing‐ review and editing.

## Ethics Statement

This study adhered to the tenets of the Declaration of Helsinki for human subjects in medical research. The study protocol was approved by the Institutional Ethics Committee of the Komfo Anokye Teaching Hospital in Ghana with registration number: KATH IRB/AP/116/21.

## Consent

The authors declare that they obtained written informed consent from the patients and/or volunteers included in the study and that this report does not contain any personal information that could lead to their identification.

## Conflicts of Interest

The authors declare that they have no known competing financial interests or personal relationships that could have influenced the work reported in this paper.

## Transparency Statement

The lead author Naa Naamuah Tagoe affirms that this manuscript is an honest, accurate, and transparent account of the study being reported; that no important aspects of the study have been omitted; and that any discrepancies from the study as planned (and, if relevant, registered) have been explained.

## Data Availability

The data that support the findings of this study are available from the corresponding author upon reasonable request.

## References

[1] Y. Sun , A. Chen , M. Zou , et al., “Time Trends, Associations and Prevalence of Blindness and Vision Loss Due to Glaucoma: An Analysis of Observational Data From the Global Burden of Disease Study 2017,” BMJ Open 12, no. 1 (2022): e053805. 10.1136/bmjopen-2021-053805 PMC8739070 34992115

[2] H. Kumar , S. Patyal , S. Singh , et al., “Analysis of Sociodemographic Profile of Glaucoma Patients With Risk Factors, Subtypes, and Disease Severity in a Tertiary Eye Care Facility in Northern India,” Indian Journal of Ophthalmology 73, no. Suppl 2 (2025): S244–S249. 39141494 10.4103/IJO.IJO_3114_23 PMC12013312

[3] R. K. Goit , “Exploring Glaucoma: From Pathogenesis to Emerging Diagnostic and Management Strategies,” Journal of Ophthalmology 9, no. 1 (2025): 8476785. 10.1155/joph/8476785 PMC12440647 40964175

[4] B. K. Belete , N. L. Assefa , A. S. Assem , and F. A. Ayele , “Determinants for Late Presentation of Glaucoma Among Adult Glaucomatous Patients in University of Gondar Comprehensive Specialized Hospital. Case‐Control Study,” PLoS One 17, no. 4 (2022): e0267582. 35486608 10.1371/journal.pone.0267582 PMC9053799

[5] M. F. Delgado , A. M. Abdelrahman , M. Terahi , et al., “Management of Glaucoma In Developing Countries: Challenges and Opportunities For Improvement,” Clinicoecon Outcomes Res [Internet] 11 (2019): 591. 31632107 10.2147/CEOR.S218277 PMC6776288

[6] A. L. Coleman and S. Miglior , “Risk Factors for Glaucoma Onset and Progression,” Survey of Ophthalmology 53, no. 6 (2008): S3–S10. 19038621 10.1016/j.survophthal.2008.08.006

[7] H. Hashemi , M. Mohammadi , N. Zandvakil , et al., “Prevalence and Risk Factors of Glaucoma in an Adult Population From Shahroud, Iran,” Journal of Current Ophthalmology 31, no. 4 (2019): 366–372. 31844784 10.1016/j.joco.2018.05.003 PMC6896457

[8] A. Le , B. N. Mukesh , C. A. McCarty , and H. R. Taylor , “Risk Factors Associated With the Incidence of Open‐Angle Glaucoma: The Visual Impairment Project,” Investigative Opthalmology and Visual Science 44, no. 9 (2003): 3783–3789. 10.1167/iovs.03-0077 12939292

[9] C. J. Siegfried and Y.‐B. Shui , “Racial Disparities in Glaucoma: From Epidemiology to Pathophysiology,” Missouri Medicine 119, no. 1 (2022): 49–54. 36033149 PMC9312450

[10] K. Grødum , A. Heijl , and B. Bengtsson , “Refractive Error and Glaucoma,” Acta Ophthalmologica Scandinavica 79, no. 6 (2001): 560–566. 11782219 10.1034/j.1600-0420.2001.790603.x

[11] D. K. Dueker , K. Singh , S. C. Lin , et al., “Corneal Thickness Measurement in the Management of Primary Open‐Angle Glaucoma,” Ophthalmology 114, no. 9 (2007): 1779–1787. 17822980 10.1016/j.ophtha.2007.04.068

[12] K. Acuff , J. H. Wu , V. Varkhedi , and S. L. Baxter , “Social Determinants of Health and Health Disparities in Glaucoma: A Review,” Clinical and Experimental Ophthalmology 52, no. 3 (2024): 276–293. 38385607 10.1111/ceo.14367 PMC11038416

[13] J. O. Sarfo , P. Mordi , E. K. Aggrey , A. S. P. Quaicoe , and P. Y. A. Attafuah , “Glaucoma Prevalence and Treatment in Sub‐Saharan Africa's Elderly Population: A Scoping Review,” BMC Geriatrics 25, no. 1 (2025): 255. 40240955 10.1186/s12877-025-05901-0 PMC12004714

[14] O. Kosoko‐Lasaki , G. Gong , G. Haynatzki , and M. R. Wilson , “Race, Ethnicity and Prevalence of Primary Open‐Angle Glaucoma,” Journal of the National Medical Association 98, no. 10 (2006): 1626–1629. 17052053 PMC2569759

[15] F. Kyari , M. Abdull , A. Bastawrous , C. Gilbert , and H. Faal , “Epidemiology of Glaucoma in Sub‐Saharan Africa: Prevalence, Incidence and Risk Factors,” Middle East African Journal of Ophthalmology 20, no. 2 (2013): 111–125. 23741130 10.4103/0974-9233.110605 PMC3669488

[16] K. T. Daba , G. G. Gessesse , J. M. Molla , and T. A. Alemu , “Assessment of Risk Factors for Advanced Open Angle Glaucoma Presentation Among Patients Visiting Jimma University Medical Center, Jimma, Ethiopia,” Ethiopian Journal of Health Sciences 32, no. 5 (2022): 929–936. 36262710 10.4314/ejhs.v32i5.8 PMC9554772

[17] M. Woodward Epidemiology: Study Design and Data Analysis. 2nd ed. Chapman & Hall/CRC Texts in Statistical Science Series. Boca Raton: Chapman & Hall/CRC; 2005.

[18] D. L. Budenz , K. Barton , J. Whiteside‐De Vos , et al., “Prevalence of Glaucoma in an Urban West African Population: The Tema Eye Survey,” JAMA Ophthalmology 131, no. 5 (2013): 651–658. 23538512 10.1001/jamaophthalmol.2013.1686 PMC4139110

[19] M. C. Leske , L. Warheit‐Roberts , and S. Y. Wu , “Open‐Angle Glaucoma and Ocular Hypertension: The Long Island Glaucoma Case‐Control Study,” Ophthalmic Epidemiology 3, no. 2 (1996): 85–96. 8841060 10.3109/09286589609080113

[20] World Health Organization , International Classification of Diseases, Tenth Revision (ICD‐10). Sixth Edition. Geneva: World Health Organization.2019

[21] R. N. Shaffer , “A Suggested Anatomic Classification to Define the Angle of the Anterior Chamber,” Transactions of the American Academy of Ophthalmology and Otolaryngology 64 (1960): 312–319.

[22] K. Omodaka , T. Kikawa , S. Kabakura , et al., “Clinical Characteristics of Glaucoma Patients With Various Risk Factors,” BMC Ophthalmology 22, no. 1 (2022): 373. 36123604 10.1186/s12886-022-02587-5 PMC9484257

[23] A. Kastner and A. J. King , “Advanced Glaucoma at Diagnosis: Current Perspectives,” Eye 34, no. 1 (2020): 116–128. 31740802 10.1038/s41433-019-0637-2 PMC7002722

[24] B. Motlagh and T. Pirbazari , “Risk Factors for Late Presentation of Chronic Glaucoma in an Iranian Population,” Oman Journal of Ophthalmology 9, no. 2 (2016): 97–100. 27433036 10.4103/0974-620X.184527 PMC4932803

[25] R. Banayot , “Clinical and Demographics Profile of Glaucoma Patients in Hebron, Palestine — A Retrospective Hospital‐Based Study,” Ophthalmology Journal 9, no. 0 (2024): 13–20.

[26] C. T. Ntim‐Amponsah , W. M. K. Amoaku , R. K. Ewusi , R. Idirisuriya‐Khair , E. Nyatepe‐Coo , and S. Ofosu‐Amaah , “Evaluation of Risk Factors for Advanced Glaucoma in Ghanaian Patients,” Eye 19, no. 5 (2005): 528–534. 15297871 10.1038/sj.eye.6701533

[27] G. Guedes , J. C. Tsai , and N. A. Loewen , “Glaucoma and Aging,” Current Aging Sciencee 4, no. 2 (2011): 110–117. 10.2174/1874609811104020110 21235491

[28] A. A. Jammal , S. I. Berchuck , A. C. Thompson , V. P. Costa , and F. A. Medeiros , “The Effect of Age on Increasing Susceptibility to Retinal Nerve Fiber Layer Loss in Glaucoma,” Investigative Ophthalmology and Visual Science 61, no. 13 (2020): 8. 10.1167/iovs.61.13.8 PMC7645210 33151281

[29] C. A. A. Hulsman , “Is Open‐Angle Glaucoma Associated With Early Menopause? The Rotterdam Study,” American Journal of Epidemiology 154, no. 2 (2001): 138–144. 11447046 10.1093/aje/154.2.138

[30] M. Rizk , A. Grise‐Dulac , and D. Gatinel , “Glaucoma in Women: What do we Know so Far ‐ A Systematic Review,” AJO International 1, no. 1 (2024): 100013.

[31] T. S. Vajaranant , S. Nayak , J. T. Wilensky , and C. E. Joslin , “Gender and Glaucoma: What We Know and What We Need to Know,” Current Opinion in Ophthalmology 21, no. 2 (2010): 91–99. 20051857 10.1097/ICU.0b013e3283360b7e PMC4326058

[32] R. C. W. Wolfs , “Genetic Risk of Primary Open‐Angle Glaucoma: Population‐Based Familial Aggregation Study,” Archives of Ophthalmology 116, no. 12 (1998): 1640–1645. 9869795 10.1001/archopht.116.12.1640

[33] M. J. S. Langman , “Systemic Hypertension and Glaucoma: Mechanisms in Common and Co‐Occurrence,” British Journal of Ophthalmology 89, no. 8 (2005): 960–963. 16024843 10.1136/bjo.2004.053397 PMC1772770

[34] Y.‐X. Zhao and X.‐W. Chen , “Diabetes and Risk of Glaucoma: Systematic Review and a Meta‐Analysis of Prospective Cohort Studies,” International Journal of Ophthalmology 10, no. 9 (2017): 1430–1435. 28944204 10.18240/ijo.2017.09.16 PMC5596230

[35] M. Nakamura , A. Kanamori , and A. Negi , “Diabetes Mellitus as a Risk Factor for Glaucomatous Optic Neuropathy,” Ophthalmologica 219, no. 1 (2005): 1–10. 15627820 10.1159/000081775

[36] T. Sato and S. Roy , “Effect of High Glucose on Fibronectin Expression and Cell Proliferation in Trabecular Meshwork Cells,” Investigative Ophthalmology and Visual Science 43, no. 1 (2002): 170–175. 11773028

[37] M. Shimmyo , A. J. Ross , A. Moy , and R. Mostafavi , “Intraocular Pressure, Goldmann Applanation Tension, Corneal Thickness, and Corneal Curvature in Caucasians, Asians, Hispanics, and African Americans,” American Journal of Ophthalmology 136, no. 4 (2003): 603–613. 14516799 10.1016/s0002-9394(03)00424-0

[38] K. Mercieca , V. Odogu , B. Fiebai , O. Arowolo , and F. Chukwuka , “Comparing Central Corneal Thickness in a Sub‐Saharan Cohort to African Americans and Afro‐Caribbeans,” Cornea 26, no. 5 (2007): 557–560. 17525651 10.1097/ICO.0b013e3180415d90

[39] M. W. Marcus , M. M. De Vries , F. G. J. Montolio , and N. M. Jansonius , “Myopia as a Risk Factor for Open‐Angle Glaucoma: A Systematic Review and Meta‐Analysis,” Ophthalmology 118, no. 10 (2011): 1989–1994.e2. 21684603 10.1016/j.ophtha.2011.03.012

[40] A. Ha , Y. K. Kim , Y. J. Park , et al., “Intraocular Pressure Change During Reading or Writing on Smartphone,” PLoS One 13 (2018): e0206061. 30359418 10.1371/journal.pone.0206061 PMC6201904

[41] H. Cho and C. Kee , “Population‐Based Glaucoma Prevalence Studies in Asians,” Survey of Ophthalmology 59, no. 4 (2014): 434–447. 24837853 10.1016/j.survophthal.2013.09.003

[42] M. B. Sultan , S. L. Mansberger , and P. P. Lee , “Understanding the Importance of IOP Variables in Glaucoma: A Systematic Review,” Survey of Ophthalmology 54, no. 6 (2009): 643–662. 19665744 10.1016/j.survophthal.2009.05.001

[43] S. Chandrasekaran , R. G. Cumming , E. Rochtchina , and P. Mitchell , “Associations Between Elevated Intraocular Pressure and Glaucoma, Use of Glaucoma Medications, and 5‐Year Incident Cataract,” Ophthalmology 113, no. 3 (2006): 417–424. 16458969 10.1016/j.ophtha.2005.10.050

[44] I.‐H. Pang and A. F. Clark , “Inducible Rodent Models of Glaucoma,” Progress in Retinal and Eye Research 75 (2020): 100799. 31557521 10.1016/j.preteyeres.2019.100799 PMC7085984

[45] V. P. Erichev , G. K. Khachatryan , and O. V. Khomchik , “Current Trends in Studying Pathogenesis of Glaucoma,” Vestnik oftal'mologii 137, no. 5 (2021): 268–274. 34669337 10.17116/oftalma2021137052268

[46] C. Dai , P. T. Khaw , Z. Q. Yin , D. Li , G. Raisman , and Y. Li , “Structural Basis of Glaucoma: The Fortified Astrocytes of the Optic Nerve Head Are the Target of Raised Intraocular Pressure,” GLIA 60, no. 1 (2012): 13–28. 21948238 10.1002/glia.21242

[47] A. Parab , S. Kavitha , A. Odayappan , and R. Venkatesh , “Clinical and Demographic Profile of Patients Less Than 40 Years of Age Presenting to Glaucoma Services at a Tertiary Care Eye Hospital in South India,” Indian Journal of Ophthalmology 70, no. 12 (2022): 4186–4192. 36453311 10.4103/ijo.IJO_963_22 PMC9940543

[48] C. T. Ntim‐Amponsah , A. Y. Seidu , V. A. Essuman , et al., “A Study of Central Corneal Thickness in Glaucoma and Nonglaucoma Patients in a West African Population,” Cornea 31, no. 10 (2012): 1093–1096. 22902491 10.1097/ICO.0b013e31823c51f7

[49] C. T. Ntim‐Amponsah , V. A. Essuman , and R. D. Edirisuriya‐Khair , “A Study of Central Corneal Thickness in Normal Ghanaians,” Nigerian Journal of Ophthalmology 15, no. 1 (2008): 1–4.

[50] N. Rahman , E. O'Neill , M. Irnaten , D. Wallace , and C. O'Brien , “Corneal Stiffness and Collagen Cross‐Linking Proteins in Glaucoma: Potential for Novel Therapeutic Strategy,” Journal of Ocular Pharmacology and Therapeutics 36, no. 8 (2020): 582–594. 32667842 10.1089/jop.2019.0118

[51] A. Mamidipaka , A. Shi , R. Lee , et al., “Socioeconomic and Environmental Factors Associated With Glaucoma in an African Ancestry Population: Findings From the Primary Open‐Angle African American Glaucoma Genetics (POAAGG) Study,” Eye 39, no. 6 (2024): 1086–1092. 39663397 10.1038/s41433-024-03470-x PMC11978978

[52] G. Gramer , B. H. F. Weber , and E. Gramer , “Migraine and Vasospasm in Glaucoma: Age‐Related Evaluation of 2027 Patients With Glaucoma or Ocular Hypertension,” Investigative Opthalmology and Visual Science 56, no. 13 (2015): 7999–8007. 10.1167/iovs.15-17274 26720447

[53] C. Xu , J. Li , Z. Li , et al., “Migraine as a Risk Factor for Primary Open Angle Glaucoma: A Systematic Review and Meta‐Analysis,” Medicine 97, no. 28 (2018): e11377. 29995778 10.1097/MD.0000000000011377 PMC6076184

